# Bulge oligonucleotide as an inhibitory agent of bacterial topoisomerase I

**DOI:** 10.1080/14756366.2017.1419218

**Published:** 2017-12-28

**Authors:** Zhaoqi Yang, Tuoyu Jiang, Hanshi Zhong, Yu Kang

**Affiliations:** a School of Pharmaceutical Sciences, Jiangnan University, Jiangsu, People’s Republic of China;; b Collaborative Innovation Center of Food Safety and Quality Control in Jiangsu Province, School of Food Science and Technology, Jiangnan University, Jiangsu, People’s Republic of China

**Keywords:** Bacterial topoisomerase I, inhibitor, bulge oligonucleotide

## Abstract

Bacterial topoisomerase I (Btopo I) was defined as potential target for discovery of new antibacterial compounds. Various oligonucleotides containing bulge structure were designed and synthesised as inhibitors to Btopo I in this investigation. The results of this study demonstrated that the designed oligonucleotides display high inhibitory efficiency on the activity of Btopo I and the inhibitory effect could be modulated by the amount of bulge DNA bases. The most efficient one among them showed an IC_50_ value of 63.1 nM in its inhibition on the activity of Btopo I. In addition, our studies confirmed that the designed oligonucleotide would induce irreversible damages to Btopo I and without any effects occur to eukaryotic topoisomerase I. It is our hope that the results provided in these studies could provide a novel way to inhibit Btopo I.

## Introduction

DNA topoisomerases I are important enzymes that release the topological stress of supercoiled DNA generated by replication and transcription and several other cellular processes^1^^,^^2^. During the course of their action, these enzymes catalyse topological rearrangements of DNA through sequential single-stranded breakage in duplex DNA, strand free rotation, and then resealing of DNA phosphodiester backbone^3–6^. They have essential regulatory functions in many biological processes, and play vital roles for cell growth and proliferation^6–8^. DNA topoisomerases I are further classified as topoisomerase IA (prokaryotic topoisomerase) and topoisomerase IB (eukaryotic topoisomerase) depend on different reacting substrates and mechanism of working^9^. Bacterial topoisomerase I (Btopo I), belongs to topoisomerase IA, is prokaryotic topoisomerase. It has been demonstrated in the past years that Btopo I is a molecular target of quinolones which have been used for antibacterial treatment in clinic^10–13^. Therefore, it is clear to us that Btopo I performs a significant role in discovery of new antibacterial agents^14–17^.

It is well known to us that negative supercoiled structures of DNA in theory are the only endowed substrate of Btopo I in organisms. However, Wang and co-worker have discovered that Btopo I can relax positive supercoiled DNA which containing a single-stranded loop^18^. Besides negative or positive supercoiled entities, certain non-supercoiled duplex oligonucleotides have been confirmed in the past decades that they could act as reacting substrates of eukaryotic topoisomerase I in some limited cases^19–23^. Further investigations revealed that when gap, nick-containing, C3-spacer-containing, and so on, were constructed into the non-supercoiled substrates, the resultant duplex structures could form covalent linkages with topoisomerase I in irreversible fashions^24–29^. In addition, certain non-supercoiled DNA was designed in our previously studies that displayed high inhibition on the activity of human topoisomerase I according to the recognise characteristic of the enzyme^30–33^. These previous studies have inspired us to speculate that the non-supercoiled oligonucleotides, if modified properly in its structure (such as introduced into a bulge structure), could serve as an inhibitor of Btopo I in the relaxation reaction of supercoiled DNA. With the aim of looking for a new type of agents beyond the chemical class of organic compounds for disturbing the action of Btopo I, we have recently examined the possibility of using non-supercoiled duplex oligonucleotides as irreversible inhibitors of this enzyme. Hence, a series of double-stranded oligonucleotides with variations in the number of bulge base are designed and examinations of its inhibitory effect on the activity of Btopo I in its relaxation of negatively supercoiled DNA are investigated. We believe such studies can serve as a basis for further design and optimisation of oligonucleotides for antibacterial application.

## Materials and methods

*Reagents*: Plasmid DNA pUC19 and bacterial topoisomerase I (*E. coli* topoisomerase I) were purchased from New England Biolabs (Ipswich, MA). Eukaryotic topoisomerase I was purchased from Takara Bio (Dalian, China). Single strand oligonucleotides were purified by HPLC and provided by Shanghai Generay Biotech Co., Ltd (China).

*Preparations of 84 bp duplex oligonucleotide*: In a solution containing two single strand oligonucleotides, 50 mM NaCl was kept at 90 °C for 10 min followed by allowing it to cool down to room temperature over a period of 1 h.

*Reactions of Btopo I with pUC 19 and designed duplex oligonucleotide*: A mixture containing 50 mM potassium acetate, 20 mM Tris-acetate, 100 µg/ml bovine serum albumin (BSA), 10 mM magnesium acetate, 250 ng pUC 19, 1 U of Btopo I, and each designed duplex oligonucleotide was prepared, respectively, and further incubated at 37 °C for 30 min. After incubation, the product was analysed by 1% agarose gel electrophoresis. The DNA bands were captured using Gel Documentation System (G:Box HR, Syngnene, Cambridge, UK).

*Reactions of eukaryotic topoisomerase I with pUC 19 and designed duplex oligonucleotide*: A mixture containing 72 mM potassium chloride, 35 mM Tris-acetate (pH 8.0), 5 mM magnesium chloride, 5 mM dithiothreitol (DTT), 5 mM spermidine, 0.01% BSA, 250 ng pUC 19, 1 U of eukaryotic topoisomerase I, and each designed duplex oligonucleotide was prepared, respectively, and further incubated at 37 °C for 30 min. After incubation, the product was analysed by 1% agarose gel electrophoresis. The DNA bands were captured using Gel Documentation System (G:Box HR, Syngnene, Cambridge, UK).

## Results and discussion

**Figure 1.**
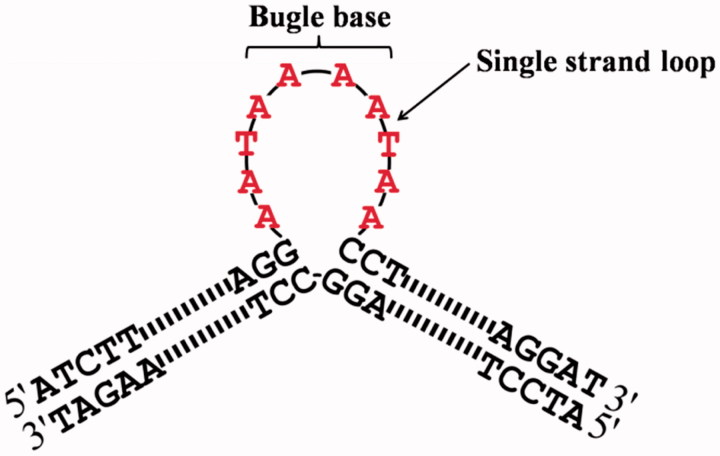
Schematic representation of designed bulge oligonucleotide.

A series of duplex non-supercoiled oligonucleotides containing bulge loop were accordingly designed and constructed during our investigations. The designed bulge oligonucleotide which was introduced the single strand loop into the duplex structure has been illustrated in Figure 1. All sequences of oligonucleotides are shown in Table 1, 84-mer single strand **B-0**, respectively, annealing with others 94-mer single strand **1**, **2**, **3**, **4**, **5**, **6** to obtain bulge oligonucleotide **B-1–1**, **B-1–2**, **B-1–3**, **B-1–4**, **B-1–5**, **B-1–10** containing different length of single strand loop. Six 84 bp duplex oligonucleotides have the same base composition, but including different numbers of bulge base. In order to ensure duplex oligonucleotides with same base composition, from **1** to **6** (Table 1), the total bases of shadowed tract and underlined tract in each oligonucleotide have same number and same base kinds. In addition, except shadowed tract and underlined tract, the rest part contains same sequence. Most importantly, it is known that the indispensable structure for Btopo I binding is a free single strand DNA^18^^,^^19^. It has been reported that the enzyme could bind and cleave positive supercoiled DNA which containing a single-stranded loop besides its innate substrate of negative supercoiled DNA. As shown in Figure 1, single strand loop which would be induced by bulge bases was introduced into the duplex oligonucleotide in middle side. Consequently, Btopo I could bind to the designed oligonucleotide and further cause an incision in one of the two duplex strands when they interact with each other. After a nick reaction is performed in designed oligonucleotide by the enzyme, religation between the cut fragments might not be able to occur properly because bulge base without corresponding base to match in opposite strand. As a result, religation cannot generate leading to the resultant duplex structures form covalent linkages with Btopo I in irreversible fashion.

**Table 1.** The sequences of oligonucleotides used in this studied.

ssDNA	Sequence
**B-0**	5′ATCCTCACCTGCATCCTGAACCCATTGACTCCCAGCGATAGGCCTTGATGTCGATAGCATTGCACGGGTCTTGTTCGATAAGAT3′
**1**	5′AATAATCTTATCGAACAAGACCCGTGCAATGCTATCGACATCAAGGACCTATCGCTGGGAGTCAATGGGTTCAGGATGCAGGTGAGGATAATAA3′
**2**	5′AATAAATCTTATCGAACAAGACCCGTGCAATGCTATCGACATCAAGGATCCTATCGCTGGGAGTCAATGGGTTCAGGATGCAGGTGAGGATAAA3′
**3**	5′AATAATCTTATCGAACAAGACCCGTGCAATGCTATCGACATCAAGGATACCTATCGCTGGGAGTCAATGGGTTCAGGATGCAGGTGAGGATAAA3′
**4**	5′AATAATCTTATCGAACAAGACCCGTGCAATGCTATCGACATCAAGGATAACCTATCGCTGGGAGTCAATGGGTTCAGGATGCAGGTGAGGATAA3′
**5**	5′AATAATCTTATCGAACAAGACCCGTGCAATGCTATCGACATCAAGGAATAACCTATCGCTGGGAGTCAATGGGTTCAGGATGCAGGTGAGGATA3′
**6**	5′ATCTTATCGAACAAGACCCGTGCAATGCTATCGACATCAAGGAATAAAATAACCTATCGCTGGGAGTCAATGGGTTCAGGATGCAGGTGAGGAT3′

The shadowed tract in duplex denotes the bulge loop.

**Figure 2.**
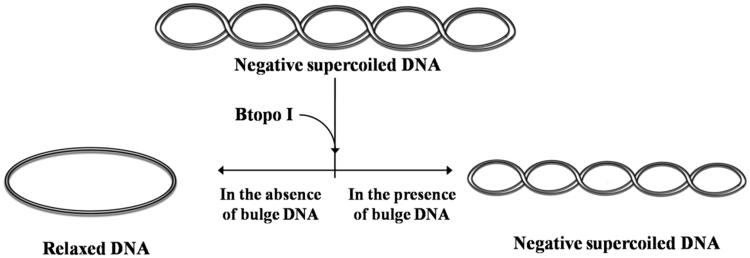
Illustration of the supercoiled pUC 19 are catalysed by Btopo I in the presence or absence of bulge DNA.

**Figure 3.**
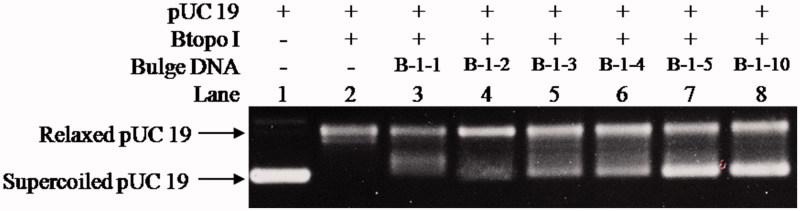
Determining the inhibitory effects to pUC 19 relaxation from **B-1–1**, **B-1–2**, **B-1–3**, **B-1–4**, **B-1–5**, and **B-1–10**. The concentration of bulge DNA was kept constant at 70 nM in all of the assay mixtures if added. The mixture containing 50 mM KAc, 20 mM Tris-Ac, 10 mM Mg(Ac)_2_, 100 μg/ml BSA (pH 7.9 at 25° C), 250 ng pUC 19, 1 U of Btopo I, and the mixtures were incubated at 37 °C for 30 min before loading on agarose gel.

With the purpose of examining whether the bulge loop structure could act as inhibitor of Btopo I, each of the six designed oligonucleotides were applied to a pUC 19 relaxing assay. In the following experiment, negative supercoiled pUC 19 plasmid is used as the substrate of Btopo I, bulge oligonucleotides were added into the system as an inhibitor to the enzyme. The relaxation levels of pUC 19 were used to characterise the enzymatic activities in the absence or presence of bulge DNA^34^. As shown in Figure 2, in the absence of oligonucleotides with bulge structure (in the left side), supercoiled DNA become nearly complete relaxation under catalysing of Btopo I. On the contrary, in the right side, relaxation of pUC 19 will be significantly retarded owing to bulge DNA is used as potential inhibitor to Btopo I. The supercoiled plasmid and relaxed pUC 19 catalysed by Btopo I are denoted in Figure 3. Lane 1 and lane 2 in Figure 3, as control experiment, are supercoiled pUC 19 and relaxed pUC 19 which is induced by Btopo I, respectively. Variation in relaxation percentage of pUC 19 represents the inhibitory efficiency of bulge DNA. The assay results were observed in the presence of 70 nM each bulge DNA. Inhibition of **B-1–1**, **B-1–2**, **B-1–3**, **B-1–4**, **B-1–5,** and **B-1–10** are shown from lane 3 to lane 9 accordingly in Figure 3. Fewer relaxation of pUC 19 is displayed in the case of **B-1–10** (land 8 in Figure 3). The diminution of relaxed pUC 19 indicates the inhibition of Btopo I by the designed bulge DNA. As shown in Figure 3, the attenuation magnitudes of Btopo I activities are gradually suppressed when the length of single-stranded loop is increased from **B-1–1** to **B-1–10** by adding the amount of bulge base. The above observations are the indications that the designed oligonucleotides could indeed reduce the activity of Btopo I and act as an inhibitor of this enzyme in the relaxation reaction of negatively supercoiled DNA.

**Figure 4.**
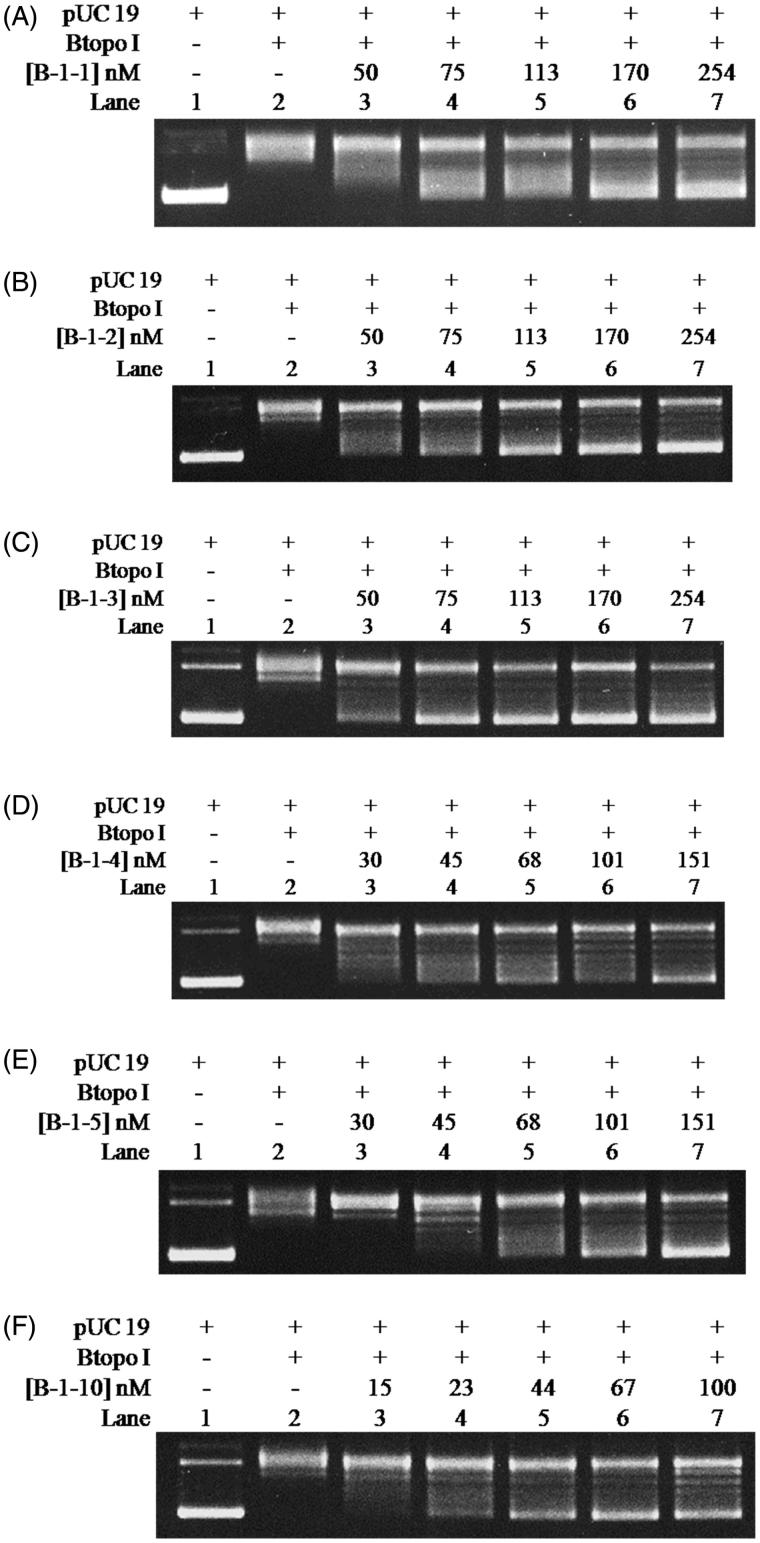
pUC 19 relaxation assay for the inhibitory effects from **B-1–1** (A), **B-1–2** (B), **B-1–3** (C), **B-1–4** (D), **B-1–5** (E), and **B-1–10** (F). Assay mixture containing 50 mM KAc, 20 mM Tris-Ac (pH 8.0), 10 mM Mg(Ac)_2_, 100 µg/ml BSA, 250 ng pUC 19, 1 U of Btopo I, and each bulge oligonucleotides were incubated at 37° C for 30 min before loading on agarose gel.

The correlation between the inhibitory efficiency and the quantity of bulge base is further explored by determining the IC_50_ of each designed bulge oligonucleotides to the activities of Btopo I catalyse negative supercoiled DNA. The relaxation of pUC 19 in the presence of a series of different concentrations of bulge DNA **B-1–1**, **B-1–2**, **B-1–3**, **B-1–4**, **B-1–5**, and **B-1–10** was monitored by agarose gel, as shown in Figure 4(A)–(F). It is easy to find that the relaxation efficiency of Btopo I decreased with the increase in concentration of each oligonucleotide in Figure 4. Inhibition percentages of Btopo I by bulge oligonucleotides are plotted against the logarithms of duplex DNA concentration and are fitted to the sigmoid curve. IC_50_ is quantified as the concentration of bulge oligonucleotides at which 50% of Btopo I activities are inhibited. As shown in Table 2, IC_50_ of all the bulge duplexes under this circumstance reach submicromolar, while that of **B-1–10** is no more than 65 nM (Figure S1 for detailed to calculate the IC_50_ in Supplementary information). These low micromolars IC_50_ suggest that the bulge oligonucleotides could indeed reduce the activity of Btopo I and act as potent inhibitor of this enzyme. Furthermore, consistent with the phenomenon in Figure 3, the values of IC_50_ decrease inversely to the increasing length of the single-stranded loop (bulge loop). **B-1–1** containing least bulge base (shortest single-stranded loop) displays the minimum inhibitory effect and the largest IC_50_ as 233.2 nM, approximate five times as that of the most bulge base duplex oligonucleotide, **B-1–10**. The above situations indicate that duplex oligonucleotides with more bulge base exhibit effective suppression on Btopo I activity. Strong suppression of Btopo I activity in presence of more bulge base duplex may result from the preferential binding of the enzyme to the bulge loop. The results from these studies revealed that the length of bulge loop in designed oligonucleotides is crucial factor for the inhibitory action of Btopo I and the activities of this enzyme can be modulated by the length of free single strand in duplex oligonucleotides.

**Table 2.** IC_50_ of bulge DNA on the inhibition of Btopo I are quantified by the relaxation assay in Figure 4(A)–(F).

Bulge	IC_50_ (nM)
B-1-1	233.2
B-1-2	137.0
B-1-3	111.7
B-1-4	91.2
B-1-5	86.4
B-1-10	63.1

Figure S1 for detailed to calculate the IC_50_ in Supplementary data.

**Figure 5.**
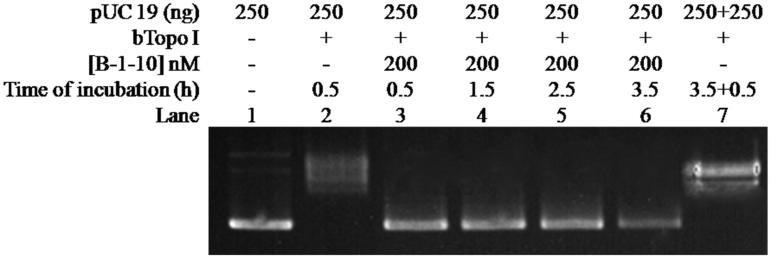
Effects of reaction time on pUC 19 relaxation assay in the presence of **B-1–10**. The concentration of **B-1–10** was kept constant at 200 nM in all of the assay mixtures if added. Assay mixtures containing 50 mM KAc, 20 mM Tris-Ac (pH 8.0), 10 mM Mg(Ac)_2_, 100 µg/ml BSA, 250 ng pUC 19, 1 U of Btopo I, and each mismatch oligonucleotides were incubated at 37 °C for 0.5 h to 3.5 h from lane 2 to lane 6. In lane 7, additional 250 ng pUC 19 was added and 0.5 h reaction time was prolonged after the reaction reach 3.5 h.

**Figure 6.**
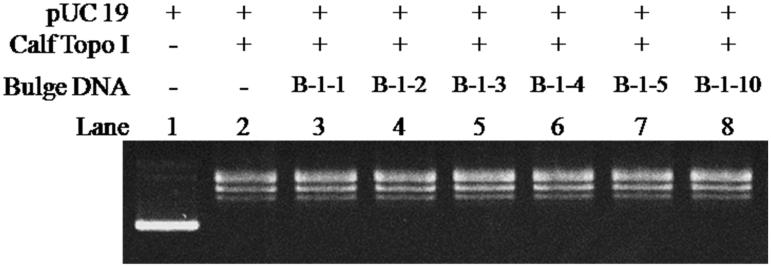
The relaxation assay was catalysed by Calf Topoisomerase I in the presence of different bulge DNA. The assay mixture containing 35 mM Tris-HCl (pH 8.0), 72 mM KCl, 5 mM MgCl_2_, 5 mM DTT, 5 mM spermidine, 0.01% BSA, 250 ng pUC 19, 1 U calf Topoisomerase I, and each mixture was incubated at 37 °C for 30 min before loading on agarose gel.

In order to further exploring whether bulge DNA could induce irreversible inactivation to Btopo I, new assay was designed to determine the inhibitory irreversibility of bulge DNA. In new assay, oligonucleotide **B-1–10** was applied to pUC 19 relaxing assay alone and the time of relaxing assay was lengthened. As shown in Figure 5, with the increasing of incubation time from 0.5 to 3.5 h (from lane 3 to lane 6), pUC 19 were still in negative supercoiled status in each reaction. None pUC 19 converted to relaxed status by reason of the time of reaction was prolonged. On the other hand, in the absent of **B-1–10**, almost all pUC 19 became relaxed status (lane 7) even after the reaction time reach 3.5 h and an extra 250 ng pUC 19 was load in the reaction mixture. It is speculate base on the above results that Btopo I nick the single-stranded loop and then not be able to splicing between these slice fragments properly due to the bugle base without corresponding base for complementation, resulting in irreversible damages to the enzyme. The above observations indicate that bulge DNA could serve as an irreversible inhibitor. In addition, with the purpose to identify bulge oligonucleotides as specific inhibitor for Btopo I, eukaryotic topoisomerase I (Calf Topoisomerase I) was utilised to study the inhibition of bugle oligonucleotides to the enzyme. As seen in Figure 6, counter to Figure 3, all pUC 19 reach relaxed situation from lane 3 to lane 8 under the same circumstance of incubation. The phenomenon turned out that these bulge oligonucleotides without observable inhibitory effects on eukaryotic topoisomerase I. In another word, bulge oligonucleotides could selective target Btopo I to inhibit its activity and exhibited no reaction to eukaryotic topoisomerase I.

## Conclusions

In conclusion, various types of duplex oligonucleotides with bulge structure were designed and synthesised in this studies, which exhibited high inhibitory efficiency on the activity of Btopo I, especially **B-1–10** (IC_50_ was 63.1 nM). Moreover, the data of assay illustrated that more potent inhibition could be found with appropriate prolonging of bulge loop structure. The results based on our research suggested that these serious of designed bulge oligonucleotides could serve as a kind of irreversible inhibitor to Btopo I. In addition, the designed substrates would possess specific targeting on Btopo I and have no any reaction with eukaryotic topoisomerase I. The investigation here demonstrates the possibility of bulge oligonucleotide to become a new therapeutic agents targeting Btopo I. Although oligonucleotide is difficult to penetrate into bacteria in current condition, we believe that it has possible to facilitate the transfection of oligonucleotide into bacteria accompany with the advancement of nano-technology and pharmaceutics. It is our expectation that the outcomes provided in these investigations could benefit the future design and optimisation of some new DNA-based therapeutical agents which target Btopo I for antibacterial treatment.

## Disclosure statement

No potential conflict of interest was reported by the authors.

## Supplementary Material

IENZ_1419218_Supplementary_Material.pdf
