# Supplementary material for: Bulge oligonucleotide as an inhibitory agent of bacterial topoisomerase I
Source: J Enzyme Inhib Med Chem. 2017 Dec 28;33(1):319–23. doi: 10.1080/14756366.2017.1419218 (PMC6009931; doi:10.1080/14756366.2017.1419218)
Supplement: IENZ_1419218_Supplementary_Material.pdf [file IENZ_A_1419218_SM7285.pdf]

Supplementary Information

Bulge Oligonucleotide as an Inhibitory Agent of Bacterial Topoisomerase I

Zhaoqi Yang[1,2,*], Tuoyu Jiang[2], Hanshi Zhong[2] and Yu Kang[2]

[1]School of Pharmaceutical Sciences, Jiangnan University, Jiangsu 214122, China
[2]Collaborative innovation center of food safety and quality control in Jiangsu Province, School of food science and technology, Jiangnan University, Jiangsu 214122, China

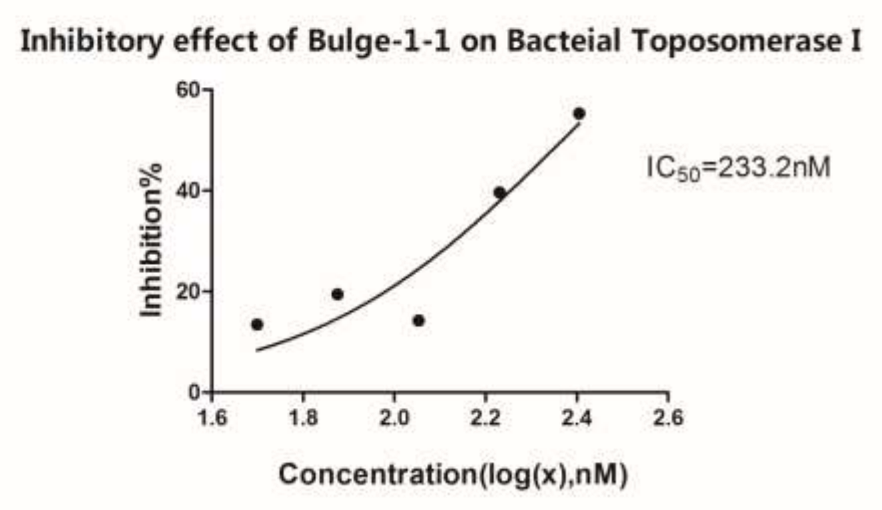

(A)

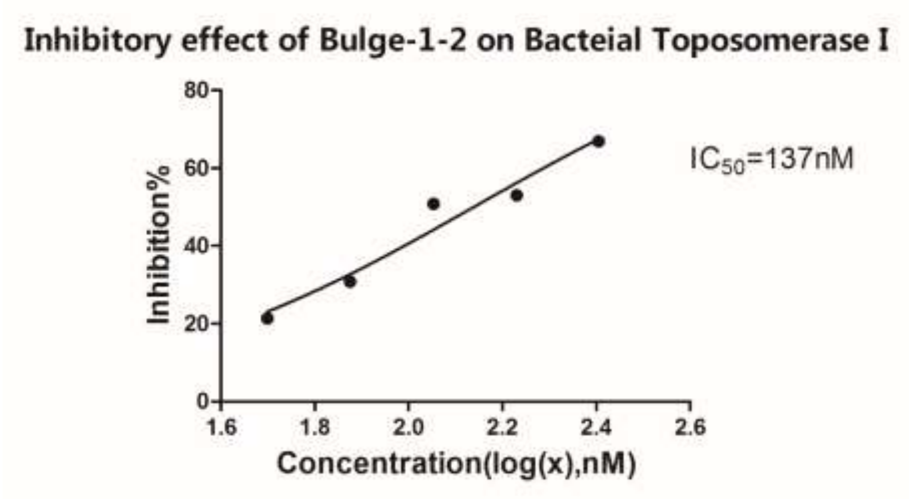

(B)

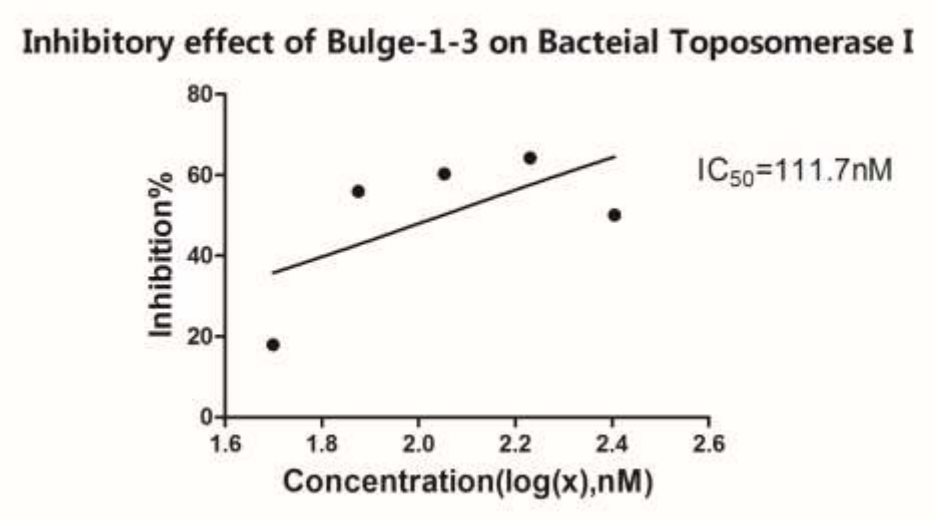
Inhibitory effect of Bulge-1-3 on Bacteial Toposomerase I
IC50=111.7nM
Inhibition%
Concentration(log(x),nM)
80
60
40
20
0
1.6
1.8
2.0
2.2
2.4
2.6

(C)

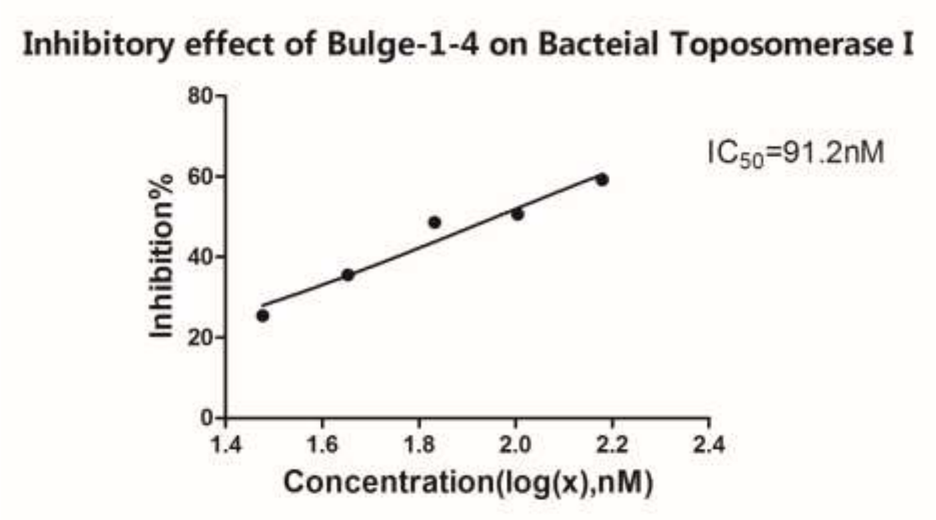
Inhibitory effect of Bulge-1-4 on Bacteial Toposomerase I
IC50=91.2nM
Inhibition%
Concentration(log(x),nM)
80
60
40
20
0
1.4
1.6
1.8
2.0
2.2
2.4

(D)

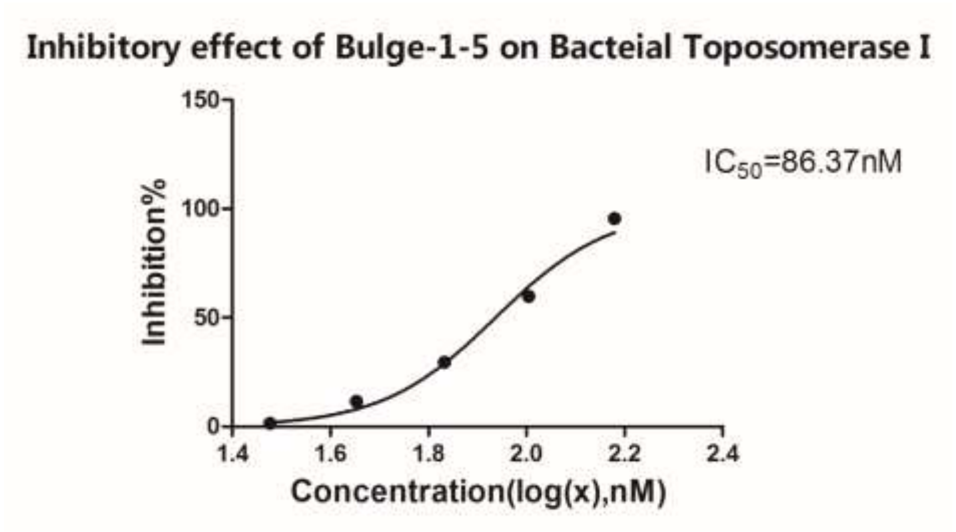

(E)

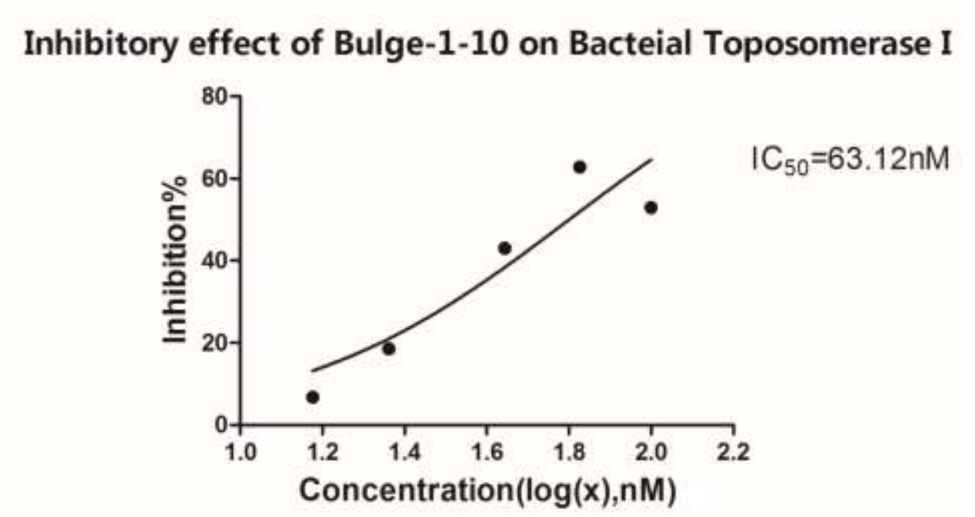

Figure S1. Correlations between concentration of **B-1-1** (A), **B-1-2** (B), **B-1-3** (C), **B-1-4** (D), **B-1-5** (E), **B-1-10** (F) and the corresponding inhibitory effects on Btopo I. Percentages of pUC 19 relaxation were defined as the ratio of band density of relaxed DNA over the some of relaxed DNA plus supercoiled DNA [relaxed DNA/ (relaxed DNA + supercoil DNA)].[1] The DNA bands were quantified using Gel Documentation System (G:Box HR, Syngnene, Cambridge, UK) equipped with Gene Tools Software.

References for Supplementary Information:

1. J. P. Laine, P. L. Opresko, F. E. Indig, J. A. Harrigan, C. von Kobbe and V. A. Bohr, *Cancer Res*, 2003, **63**, 7136-7146.
